# Implementation of web-based open-source radiotherapy delineation software (WORDS) in organs at risk contouring training for newly qualified radiotherapists: quantitative comparison with conventional one-to-one coaching approach

**DOI:** 10.1186/s12909-021-02992-2

**Published:** 2021-11-08

**Authors:** Adams Hei Long Yuen, Alex Kai Leung Li, Philip Chung Yin Mak, Hin Lap Leung

**Affiliations:** Oncology Centre, St. Teresa’s Hospital, 327 Prince Edward Road, Hong Kong Special Administrative Region, China

**Keywords:** Organs at risk, Contouring, Web-based training, One-to-one training, Medical dosimetry, Radiotherapy, Manpower

## Abstract

**Background:**

Due to the role expansion of radiotherapists in dosimetric aspect, radiotherapists have taken up organs at risk (OARs) contouring work in many clinical settings. However, training of newly qualified radiotherapists in OARs contouring can be time consuming, it may also cause extra burden to experienced radiotherapists. As web-based open-source radiotherapy delineation software (WORDS) has become more readily available, it has provided a free and interactive alternative to conventional one-to-one coaching approach during OARs contouring training. The present study aims to evaluate the effectiveness of WORDS in training OARs contouring skills of newly qualified radiotherapists, compared to those trained by conventional one-to-one coaching approach.

**Methods:**

Nine newly qualified radiotherapists (licensed in 2017 – 2018) were enrolled to the conventional one-to-one coaching group (control group), while 11 newly qualified radiotherapists (licensed in 2019 – 2021) were assigned to WORDS training group (measured group). Ten OARs were selected to be contoured in this 3-phases quantitative study. Participants were required to undergo phase 1 OARs contouring in the beginning of the training session. Afterwards, conventional one-to-one training or WORDS training session was provided to participants according to their assigned group. Then the participants did phase 2 and 3 OARs contouring which were separated 1 week apart. Phase 1 – 3 OARs contouring aimed to demonstrate participants’ pre-training OARs contouring ability, post-training OARs contouring ability and knowledge retention after one-week interval respectively using either training approach. To prevent bias, the computed tomography dataset for OARs contouring in each phase were different. Variations in the contouring scores for the selected OARs were evaluated between 3 phases using Kruskal-Wallis tests with Dunn tests for pairwise comparisons. Variations in the contouring scores between control and measured group in phase 1 – 3 contouring were analyzed using Wilcoxon signed-rank test. A *p*-value < 0.05 was considered to be statistically significant.

**Results:**

In both control group and measured group, significant improvement (*p* < 0.05) in phase 2 and 3 contouring scores have been observed comparing to phase 1 contouring scores. In comparison of contouring scores between control group and measured group, no significant differences (*p* > 0.05) were observed in all OARs between both groups.

**Conclusions:**

The results in this study have demonstrated that the outcome of OARs contouring training using WORDS is comparable to the conventional training approach. In addition, WORDS can offer flexibility to newly qualified radiotherapists to practice OARs contouring at will, as well as reduce staff training burden of experienced radiotherapists.

## Introduction

Accurate delineation of target volumes and organs at risk (OARs) are critical for maximising tumour control and minimising radiation toxicities [1]. Recent advances in computing power, algorithms and big data collection are resulting in the application of artificial intelligence in radiotherapy [2]. There is no doubt that automatic image segmentation, including atlas based (ABAS) and deep learning based (DLAS) autosegmentation, will play a critical role in the future of clinical radiotherapy planning, particularly to OARs contouring. However, a previous study has also reported that the accuracy of ABAS is highly dependent on the similarity of the atlas and the underlying patient, the inaccurate delineation may result in time-consuming manual postprocessing [3]. In DLAS, the limitation is that the algorithm is simply learning from clinical data, which includes multiple observer preferences and possible imperfections [4, 5]. Therefore, accurate contouring of OARs is still extensively relied on manual based OARs contouring in many clinical practices.

Variability in contouring is deemed to be one of the greatest sources of error in medical dosimetry [6]. Previous studies have stated that variations in contouring can be occurred in health professionals with different level of experience [7–9]. Variations in contouring, as a result of knowledge deficit, can compromise the reliability of dosimetric comparison of radiotherapy treatment plans [10]. Variations in contouring have also been found to decrease overall survival and local control [11]. Consequently, poor quality radiotherapy can cause detrimental effect to the anticipated treatment efficacy [7]. Due to the importance of accurate manual based contouring or post-processing, the provision of adequate training in OARs contouring to newly qualified radiotherapists is crucial. However, training of newly qualified radiotherapists in OARs contouring can be time consuming, in which it may cause extra burden to experienced radiotherapists in addition to the heavy clinical workload. It is even practically impossible in busy clinical settings.

Web-based open-source radiotherapy delineation software (WORDS), such as eContour [12], EduCase [13], Anatom-e [14], and ProKnow™ Contouring Accuracy [15], are interactive online platforms that participants can use to study and practice OARs contouring against a practice set. As WORDS has become more readily available, the application of WORDS has provided a free and interactive alternative to conventional contouring reference aids, such as consensus guidelines or textbooks. Previous randomized trials have also shown that WORDS is effective alternative to traditional didactic lectures aiming at teaching contouring skills in medical students and residents [16–19]. Due to the role expansion of radiotherapists in dosimetric aspect [20, 21], the effectiveness of WORDS in training newly qualified radiotherapists’ OARs contouring skills should also be studied.

The present study aims to evaluate the effectiveness, using quantitative data, of WORDS in training OARs contouring skills of newly qualified radiotherapists, compared to those trained by conventional one-to-one coaching approach.

## Methodology

### Study design

All newly qualified radiotherapists in St. Teresa’s Hospital from 2017 to 2021 were enrolled in the present study. All participants did not have post-employment clinical experience in medical dosimetry, including organs at risk (OARs) contouring and treatment planning. Nine newly qualified radiotherapists (licensed in 2017 – 2018) were enrolled to the conventional one-to-one coaching group (control group), while 11 newly qualified radiotherapists (licensed in 2019 – 2021) were enrolled to web-based open-source radiotherapy delineation software (WORDS) training group (measured group).

**Fig. 1:**
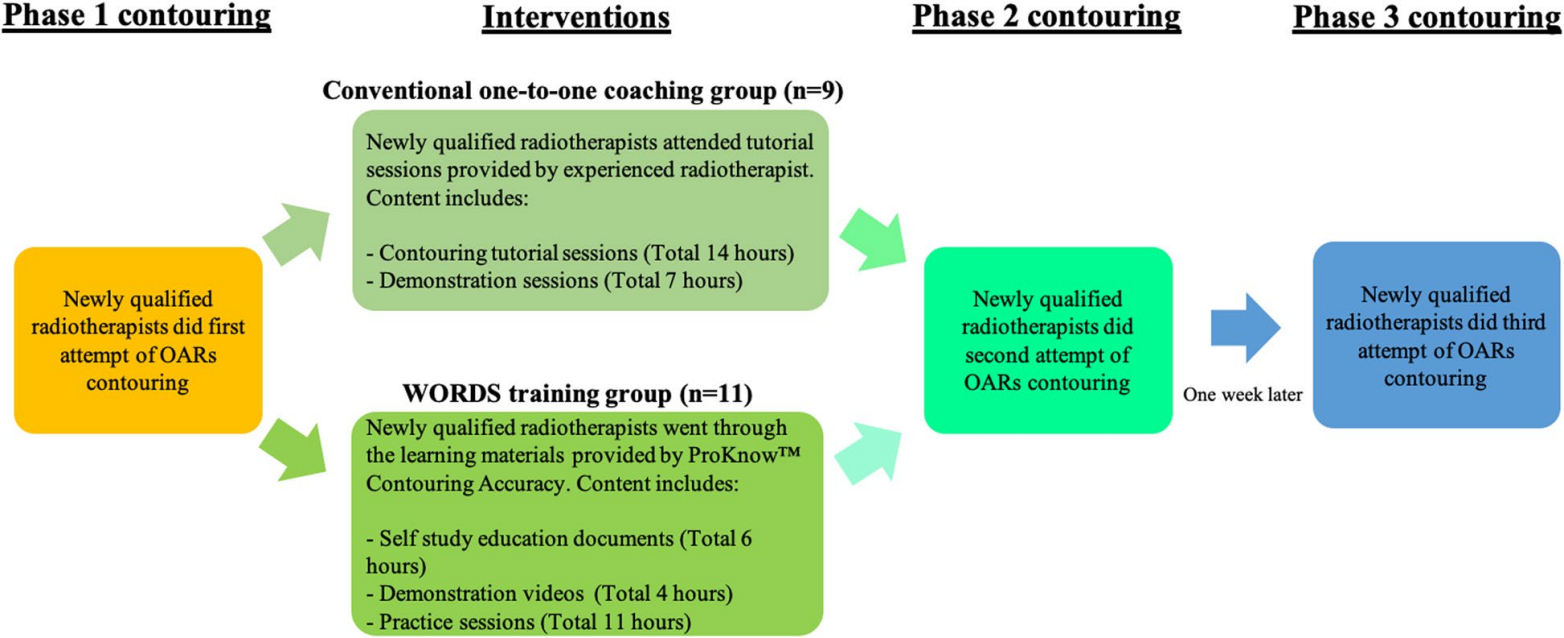
Study schema

The present study used a 3-phases quantitative analysis design (the study schema was shown in Fig. 1). Phase 1 contouring aimed to demonstrate participants’ pre-training OARs contouring ability. Phase 2 contouring aimed to demonstrate participants’ post-training OARs contouring ability. Phase 3 contouring aimed to demonstrate participants’ knowledge retention using either training approach. To prevent bias, the computed tomography (CT) dataset for OARs contouring in each phase were different.

### Contour platform and organs at risk (OARs) selection

All contours in control group and measured group were made on a WORDS - ProKnow™ Contouring Accuracy (ProKnow™ Systems, Sanford, FL, USA), in which it has been specifically designed for quality anatomical contouring. Ten OARs were selected: brainstem, cochlea, optic nerve, parotid gland, submandibular gland, bladder, rectum, penile bulb, seminal vesicle and prostate. Number of segmentations in each OARs CT dataset were included in Table 1. Participating newly qualified radiotherapists were required to contour all the aforementioned OARs. Participants were allowed to use all available contouring functions provided by ProKnow™ Contouring Accuracy during OARs contouring.

**Table 1:** Number of segmentations in CT data for OARs contouring provided by ProKnow™ Contouring Accuracy

Selected organs at risk	Number of segmentations (slice thickness) in CT data provided by ProKnow™ Contouring Accuracy		
	Phase 1 contouring	Phase 2 contouring	Phase 3 contouring
Brainstem	31 (2 mm)	24 (2.5 mm)	54 (1 mm)
Cochlea	4 (2 mm)	4 (2 mm)	6 (1 mm)
Optic nerve	5 (2 mm)	3 (2 mm)	20 (1 mm)
Parotid gland	32 (2 mm)	28 (2 mm)	27 (2.5 mm)
Submandibular gland	25 (2 mm)	19 (2 mm)	20 (2.5 mm)
Bladder	30 (2 mm)	23 (2.5 mm)	55 (2 mm)
Rectum	34 (2.5 mm)	77 (1.5 mm)	74 (1.5 mm)
Penile bulb	5 (3 mm)	4 (3 mm)	12 (1.5 mm)
Seminal vesicle	9 (3 mm)	9 (3 mm)	10 (1.5 mm)
Prostate	20 (3 mm)	15 (3 mm)	25 (1.5 mm)

### Scoring

The accuracy of OARs contouring was quantified by contour assessment system StructSure™ accuracy score [22] (US Patent 8,081,813) provided by the in-built system of ProKnow™ Contouring Accuracy. StructSure™ accuracy score has provided a quantitative analysis of contouring accuracy that compares participant’s contouring with expert contouring (gold standard provided by the in-built system). The contouring scores (range from 0 to 100) were calculated as:

$$100\times \frac{Number\ of\ expert\ voxels- Sum\ of\ penalties\ over\ all\ voxels}{Number\ of\ expert\ voxels.}$$

**Fig. 2:**
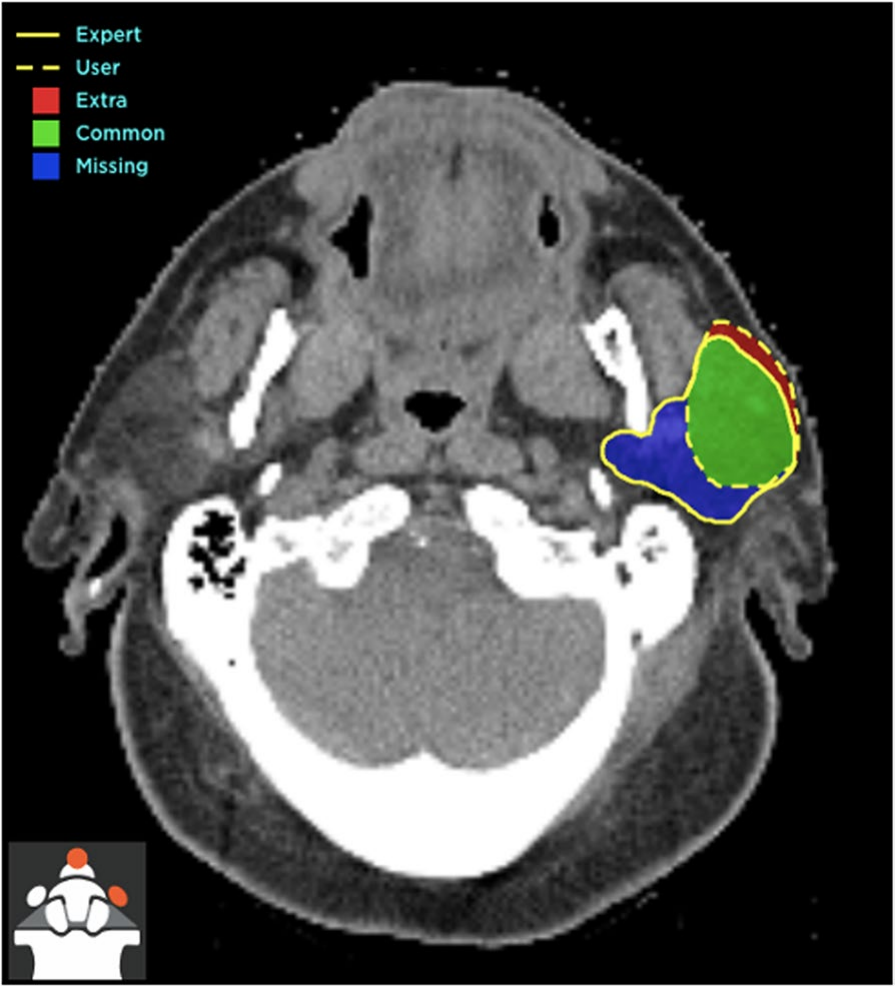
Figure illustrating missing contour, extra contour and matching contour

Penalties are referred to errant voxels, which can be “missing contour” (participant’s contouring is smaller than the expert’s contouring) or “extra contour” (participant’s contouring is larger than the expert’s contouring) (Fig. 2). No penalty will be given if the errant voxel is less than or equal to 1 mm. If the errant voxel is larger than 1 mm, the voxel penalty levied per errant voxel is 0.5 voxels per mm. Penalty can be calculated as:

$$0.5\ voxels/ mm\times \left( distance\ error\ to\ expert's\ contour\ (mm)-1 mm\ forgiveness\ region\right)$$

### Conventional one-to-one coaching group (control group)

The control group aimed to develop participants’ OARs contouring skills by conventional one-to-one coaching approach. Each newly qualified radiotherapist in conventional training group was assigned to an experienced radiotherapist with at least 4 years of clinical experience. The newly qualified radiotherapists in control group were invited to undergo first attempt of OARs contouring (phase 1) in the beginning of the training session. After that, participating newly qualified radiotherapists attended 3 days training sessions that was held by the assigned experienced radiotherapists. The training sessions included 14 h of tutorial sessions and 7 h of demonstration sessions. The tutorial sessions were held in form of PowerPoint based lecture courses using review articles, consensus guidelines and textbooks as reference materials [23–27]. In demonstration sessions, experienced radiotherapists demonstrated OARs contouring using clinically used CT datasets as example. Participants were allowed to practice OARs contouring after demonstration. Followed by the tutorial sessions, participants did OARs contouring on another set of CT images (phase 2). One week later, the participants did third attempt of OARs contouring (phase 3).

To simulate the conventional training approach, contouring metrics and contouring scores were not disclosed to the participants in conventional training group during phase 1 – 3 OARs contouring.

### Web-based open-source radiotherapy delineation software (WORDS) training group (measured group)

The measured group aimed to develop participants’ OARs contouring skills using WORDS. The newly qualified radiotherapists in WORDS training group were invited to undergo first attempt of OARs contouring (phase 1) in the beginning of the training session. Then, the participants went through WORDS training sessions, which included 6 h self-study of education documents, 4 h contouring demonstration video sessions and 11 h practice sessions. The education documents and contouring demonstration videos were freely provided to public by ProKnow™ Contouring Accuracy upon registration. In practice sessions, participants were allowed to practice OARs contouring using CT datasets in ProKnow™ Contouring Accuracy. All CT datasets used for practice were not included in the phase 1 – 3 OARs contouring. After WORDS training sessions, the participating newly qualified radiotherapists did OARs contouring on another set of CT images (phase 2). One week later, the participants did third attempt of OARs contouring (phase 3).

Throughout phase 1 – 3 OARs contouring, newly qualified radiotherapists in WORDS training group were allowed to review their contouring metrics, such as missing, extra and matching volume, but were blind to the contouring scores.

### Statistical analysis

In measured and control group, variations in the StructSure™ accuracy score for the selected OARs were evaluated between 3 phases using Kruskal-Wallis tests with Dunn tests for pairwise comparisons. The variations of StructSure™ accuracy score between measured and control group in phase 1-3 contouring were analyzed using Wilcoxon signed-rank test. A *p*-value < 0.05 was considered to be statistically significant. All statistical analyses were performed using SPSS version 26 statistical software (IBM, USA).

## Results

A total of 600 OARs contouring data had been collected for analysis. Results of the newly qualified radiotherapists’ contouring in each phase were summarized in Table 2 as mean ± standard deviation (SD).

**Table 2:** Contouring scores of WORDS training group and conventional one-to-one coaching group using StructSure™ accuracy score (presented as mean ± SD)

	Conventional one-to-one coaching group (control group)						WORDS training group (Measured group)					
OARs	Contouring scores (Mean ± SD)			*p*-value (Phase 1 & 2)	*p*-value (Phase 1 & 3)	*p*-value (Phase 2 & 3)	Contouring scores (Mean ± SD)			*p*-value (Phase 1 & 2)	*p*-value (Phase 1 & 3)	*p*-value (Phase 2 & 3)
	Phase 1	Phase 2	Phase 3				Phase 1	Phase 2	Phase 3			
Brainstem	58.26 ± 17.39	82.34 ± 4.76	83.3 ± 9.49	**0.028**	**0.024**	> 0.05	61.61 ± 12.56	82.56 ± 6.01	82.50 ± 8.39	**0.017**	**0.028**	> 0.05
Cochlea	29.15 ± 21.49	79.80 ± 16.03	70.21 ± 13.10	**0.009**	**0.034**	> 0.05	30.60 ± 24.68	75.55 ± 14.11	74.72 ± 14.28	**0.024**	**0.033**	> 0.05
Optic nerve	44.84 ± 26.80	79.25 ± 12.80	62.04 ± 8.94	**0.011**	**0.033**	**0.049**	43.97 ± 28.22	84.24 ± 6.25	82.49 ± 8.94	**0.017**	**0.045**	> 0.05
Parotid gland	49.60 ± 23.71	80.78 ± 11.33	82.45 ± 4.85	**0.016**	**0.013**	> 0.05	52.40 ± 17.20	83.97 ± 5.25	84.73 ± 3.55	**0.012**	**0.009**	> 0.05
Submandibular gland	26.84 ± 31.83	87.82 ± 8.07	89.92 ± 9.24	**0.040**	**0.030**	> 0.05	45.98 ± 25.17	87.00 ± 10.25	87.63 ± 9.80	**0.015**	**0.012**	> 0.05
Bladder	70.44 ± 4.48	94.95 ± 2.31	90.09 ± 4.10	**0.003**	**0.048**	> 0.05	72.08 ± 8.14	92.88 ± 2.38	91.47 ± 2.79	**0.005**	**0.021**	> 0.05
Rectum	43.06 ± 26.30	83.61 ± 11.88	83.21 ± 10.48	**0.013**	**0.028**	> 0.05	50.97 ± 22.65	82.51 ± 9.63	79.23 ± 5.10	**0.007**	**0.039**	> 0.05
Penile bulb	52.23 ± 32.19	86.00 ± 8.67	92.24 ± 5.31	**0.028**	**0.006**	> 0.05	57.80 ± 13.50	87.92 ± 7.12	91.13 ± 1.73	**0.015**	**0.007**	> 0.05
Seminal vesicle	58.70 ± 25.96	84.15 ± 5.08	81.13 ± 2.55	**0.014**	**0.040**	> 0.05	71.13 ± 5.42	84.19 ± 3.38	85.02 ± 7.50	**0.021**	**0.015**	> 0.05
Prostate	52.89 ± 21.72	84.77 ± 3.84	84.66 ± 3.67	**0.027**	**0.022**	> 0.05	55.01 ± 12.75	87.34 ± 3.49	85.17 ± 5.58	**0.007**	**0.015**	> 0.05

*p*-value < 0.05 (**bold**) was considered to be statistically significant (Kruskal-Wallis tests with Dunn tests for pairwise comparisons)

Abbreviations: *OARs* organs at risk, *WORDS* Web-based Open-source Radiotherapy Delineation Software

Comparing contouring scores between each phase, both measured and control group demonstrated significant difference between phase 1 & 2 contouring and phase 1 & 3 contouring (*p* < 0.05) in all OARs. In measured group, no significant difference (*p* > 0.05) was observed in all OARs between phase 2 & 3 contouring. No significant differences (*p* > 0.05) were observed in contouring scores between phase 2 & 3 contouring, except optic nerve, in conventional one-to-one coaching group (Table 2).

In comparison of contouring scores between WORDS training group and conventional one-to-one coaching group, no significant differences (*p* > 0.05) were observed in all OARs between both groups.

## Discussion

In the era of rapid technological advance, there has been manifold development in strategies to increase the efficacy of radiotherapy, including but not limit to calibration [28], simulation [29], OARs contouring [30], dosimetry-based planning [31], treatment [32] and quality assurance [33]. Accurate OARs contouring is deemed a critical step in the development of effective radiotherapy plans since all subsequent radiotherapy planning and delivery process are dependent on OARs contouring. Therefore, OARs contouring is considered prerequisites for achieving the optimal curative effect for patients. The emerging role of radiotherapists in dosimetric aspect has led to the need of newly qualified radiotherapists to be equipped with OARs contouring skills [21]. To the best of authors’ knowledge, the present study is the first to provide quantitative results to evaluate the effectiveness of WORDS in training OARs contouring skills of newly qualified radiotherapists compared to those trained by conventional one-to-one coaching approach.

Using conventional one-to-one coaching approach for OARs contouring training is highly dependent on the availability of the experienced radiotherapists. To participate training, newly qualified radiotherapists also need to be able to allocate the requisite time in their schedule. With time-pressures often influencing the capacity of an oncology centre to deliver training to newly qualified radiotherapists, conventional one-to-one coaching approach may not be a viable option for busy clinical settings.

Meanwhile, interobserver variability in OARs contouring could exist in experienced health professionals, even though OARs contouring guidelines are available [34]. As such, the inconsistent knowledge of OARs contouring from experienced radiotherapists might impart to newly qualified radiotherapists during conventional one-to-one coaching, continuing the contouring variability.

Previous studies have demonstrated that web-based education is highly valued by health professions trainees [35]. WORDS provides online contouring practice platforms, which allow newly qualified radiotherapists to practice OARs contouring and validate their contouring by comparing with experts’ contouring. The web-based platforms also offer flexibility to users to adapt the format and content, making OARs contouring training more learner-centric. In the present study, ProKnow™ Contouring Accuracy has been used to evaluate the effectiveness in training OARs contouring skills of newly qualified radiotherapists. This web-based open-source platform can provide contouring demonstration video and practice to users, detailed results (e.g. distance-volume histogram, matching volume, extra volume and missing volume) have also been provided for evaluation after contouring practice. Newly qualified radiotherapists can revise the contouring demonstration video and OARs contouring at will, even experienced radiotherapists cannot be physically present. To date, this platform has provided 190 image series of various organs for users to do contouring practice.

The results in the present study have demonstrated significant improvement (*p* < 0.05) in contouring scores after going through the learning materials provided by ProKnow™ Contouring Accuracy (phase 1 vs. phase 2 contouring). In this group, the contouring scores of all OARs have no significant difference (*p* > 0.05) between phase 2 and phase 3 contouring, in which the 2 phases have been separated 1 week apart. The results can indicate that knowledge retention is achievable using WORDS training approach. In comparing contouring scores between WORDS training group and conventional one-to-one coaching group, no significant differences (*p* > 0.05) have been observed in all OARs indicating that WORDS training approach is comparable to the conventional training approach. In contrast to phase 2 and 3 OARs contouring, the standard deviations are generally larger during phase 1 contouring in both groups, indicating that contouring scores are more spread in this phase. The presumable reason could be contouring error before training sessions in the present study. It is also worth highlighting that the less spread out of phase 2 and 3 contouring scores may indicate that both training approaches could similarly reduce inter-observer variability of OARs contouring. Extension of this research could examine the effectiveness of reducing inter-observer variability using both approaches in larger sample size.

Dice similarity coefficient (DSC) [36] is a common metric for measuring contouring overlap and has been frequently used to compare contour accuracy in previous studies [37, 38]. However, some studies have reported that DSC may be unfavorable to small contoured object as a few pixels of misclassification can lead to a large decrease of the coefficient, meanwhile, DSC is also not sensitive enough to large errors when the contoured object is large [39, 40]. In the present study, contouring quality of OARs between WORDS training group and conventional one-to-one coaching group has been compared using StructSure™ accuracy score. StructSure™ is a metric score calculated by volumetric quantification algorithm. OARs have been discretized into cubic voxels. Penalty per voxel is applied as a function of distance-to-agreement for errant voxels, which allow larger contouring errors to be penalized more than smaller contouring errors and, hence, provide superior sensitivity.

The present study has several limitations worth noting. First, the study population has represented newly qualified radiotherapists in single oncology centre, thus, the results might not be generalizable to other clinical settings. While OARs contouring training should be carried out for newly qualified radiotherapists on a year-by-year basis to meet the manpower demand, having a randomized sample is unlikely to happen in our oncology centre due to the low number of recruits annually. To minimize the selection bias, all participants recruited in the present study have graduated from bachelor’s degree in radiotherapy in Hong Kong and certified by local radiographers’ board. Therefore, it can be assumed that all participants have similar knowledge level in OAR contouring upon entry into the present study. Additionally, the present study has only included 10 OARs because of their complexity involved during OARs contouring. Although the present study has demonstrated that the WORDS training group can achieve comparable contouring quality in the selected OARs, it is unclear whether similar outcome would translate to other unselected OARs.

In spite of the aforementioned limitations, valuable insights have been gained in the present study. The results in the present study have demonstrated that the effectiveness of WORDS is comparable to the conventional one-to-one coaching approach in providing OARs contouring training to newly qualified radiotherapists. In addition to the comparable effectiveness, WORDS can provide flexibility to users to practice OARs contouring at will, even experienced radiotherapist cannot be physically present. WORDS is especially useful in busy clinical settings that it can reduce the burden of experienced radiotherapists to coach newly qualified radiotherapists OARs contouring. Therefore, WORDS has the potential to be considered as an alternative to the conventional one-to-one coaching approach.

## Data Availability

The data that support the findings of this study are available from the Oncology Centre, St. Teresa’s Hospital (HKSAR) but restrictions apply to the availability of these data, which were used under permission for the current study, and so are not publicly available. Data are however available from the authors upon reasonable request and with permission of the Oncology Centre, St. Teresa’s Hospital (HKSAR) at the following e-mail address: sthochk@gmail.com.
